# Retraction Note: Dissecting closely linked association signals in combination with the mammalian phenotype database can identify candidate genes in dairy cattle

**DOI:** 10.1186/s12863-018-0698-4

**Published:** 2018-12-11

**Authors:** Zexi Cai, Bernt Guldbrandtsen, Mogens Sandø Lund, Goutam Sahana

**Affiliations:** Center for Quantitative Genetics and Genomics, Department of Molecular Biology and Genetics, Aarhus University, 8830 Tjele, Denmark

**Retraction of: BMC Genet (2018) 19:30**

**https://doi.org/10.1186/s12863-018-0620-0**

This article [1] has been retracted by the authors after discovering that a set of markers which would not pass quality control criteria has been used for the association study described, resulting in false positives being included. As a result, some sections of the article are inaccurate. To avoid confusion this article has been retracted and the authors have been given the opportunity to resubmit their corrected data. All authors agree to this retraction.
